# Nitrogen Substituted Phenothiazine Derivatives: Modelling of Molecular Self-Assembling

**DOI:** 10.3390/ijms12053102

**Published:** 2011-05-12

**Authors:** Attila Bende, Ioan Turcu

**Affiliations:** Molecular and Biomolecular Physics Department, National Institute for Research and Development of Isotopic and Molecular Technologies, Donath Street, Nr. 65-103, Ro-400293 Cluj-Napoca, Romania; E-Mail: bende@itim-cj.ro

**Keywords:** nitrogen substituted phenothiazine, intermolecular interaction, dispersion effects, local perturbation method, π-stacking

## Abstract

The study aims to present a detailed theoretical investigation of noncovalent intermolecular interactions between different π–π stacking nitrogen substituted phenothiazine derivatives by applying second-order Møller-Plesset perturbation (MP2), density functional (DFT) and semiempirical theories. The conformational stability of these molecular systems is mainly given by the dispersion-type electron correlation effects. The density functional tight-binding (DFTB) method applied for dimer structures are compared with the results obtained by the higher level theoretical methods. Additionally, the optimal configuration of the investigated supramolecular systems and their self-assembling properties are discussed.

## 1. Introduction

Weak noncovalent intermolecular forces such as hydrogen bonds, π–π stacking play an important role in the formation of stable and structurally well-defined supramolecular structures [1,2]. Among the noncovalent intermolecular forces π–π stacking or aromatic-aromatic interactions in delocalized π-systems play a wide range of molecular recognition and self assembly phenomena [3,4]. It has been estimated that around 60% of aromatic side chains (histidine, phenylalanine, tyrosine, tryptophan) participate in π–π stacking interactions in proteins [5]. Intra- and inter-strand stacking interactions have been identified to be as important as hydrogen-bonding interactions for the stabilization of the DNA double helix for example (See Reference [6] and references therein). The study of the gas-phase stacking interactions between the five natural DNA and RNA nucleobases and the four aromatic amino acid residues reveals that the largest stacking interactions between the natural nucleobases and amino acids approach the strength of the weakest hydrogen-bonding (adenine–thymine interaction) in DNA [7]. All these findings suggest that stacking interactions between the natural nucleobases and aromatic amino acids likely play a much bigger role in biological processes than previously anticipated.

In spite of the fact that parallel-displaced π–π stacking interactions [8] have been recognized to be an important force in stabilizing the double-helical structure of DNA and the tertiary structure of proteins, less features are known about their roles in self-assembled monolayers (SAM). SAMs are organic assemblies formed by the absorption of molecular constituents from solution or the gas phase onto the surface of solids or in irregular arrays on the surface of liquids where the adsorbents organize spontaneously (or induced [9]) into crystalline (or semicrystalline) structures or into different ordered forms. SAMs have four essential components which define their structure: the metal substrate, the ligand (or headgroup), the spacer (usually built by alkane chain), and the terminal functional group, where the last three components form a self-consistent molecular unit [10]. The matter of self-assembly can be characterized by the spontaneous and reversible organization of these molecular units into ordered structures by noncovalent interactions which occur between the spacer units as well as between the functional groups.

In our previous work [11] we have investigated the self-assembling properties of different molecular complexes where for the functional group unit the phenothiazine molecule have been considered. We have demonstrate that the “V” stacking form of the phenothiazine dimer shows one of the strongest intermolecular interaction among the studied dimer conformations and has a large affinity to form large one dimensional molecular chains. In order to keep the high symmetry order of the molecular self-association the linker unit must follow the pattern defined by the functional group of the phenothiazine. Accordingly, we have also investigated the molecular self-association properties in case of different long alkyl chains. We have found two, characteristic configuration of the alkyl dimers, called side-parallel and top-parallel chains. The results show that in case of the alkyl dimers the side-parallel configuration is preferred. The energy difference between the side-parallel and top-parallel conformations of decane is around 3.6 kcal/mol. On the other hand, if we want to keep the high ordered structure given by the functional group, one need to have the top-parallel configuration for the linker unit. This means that, one has to find out the optimal length of the alkyl chain which is long enough to facilitate the parallel aggregation of the phenothiazine aromatic rings but is reasonably short to not allow the appearance of the distorted oligomers. We found that, adding together the attractive effects of the PTZ fragments, the constructive contributions of the top-parallel alkyl chain and of the thiol group as well as the destructive effect of the side-parallel alkyl chain we establish that the pentane chain has the optimal length.

For increasing the self-assembling capability of the organic molecules from the phenothiazine family we propose a new class of nitrogen substituted phenothiazine derivatives with a stronger tendency to self-organize in supramolecular structures. These nitrogen-substituted systems are expected to enhance stacking interactions by polarizing the aromatic ring and thereby promoting electrostatic interactions.

The π–π interactions between benzene and the aromatic nitrogen heterocyclic pyridine, pyrimidine, 1,3,5-triazine, 1,2,3-triazine, 1,2,4,5-tetrazine, and 1,2,3,4,5-pentazine were systematically investigated by the Hobza group [12]. Their conclusion was that with increasing number of nitrogen atoms in the heterocycles, the distance between the rings decreases while the binding energy increases. Moreover, all intermolecular energy components increase with increasing number of nitrogen atoms in the heterocycle. Studying the π–π interactions between stacked DNA/RNA bases, the same idea was also drawn by Mignon *et al.* [13,14], completed with the fact that the H-bonding capacity of the N and O atoms of cytosine increases linearly with the electrostatic repulsion between the stacked heterocyclic rings. On the other hand, Mishra *et al.* [15] stated that the stronger stability of double helical DNA and RNA structures is obtained by the presence of N and O atoms. In case of crystal packing Główka *et al*. [16] demonstrate that molecules containing nitrogen atom(s) in the aromatic rings show a significantly higher population of crystal structures with stacking. Heteroatoms in an aromatic system induce differentiation of (and) electron distributions; thus they influence stacking-type preferences. This is due to the fact that, in the π–π parallel stacked aromatic arrangements, displacement of the rings favors the minimization of repulsive electrostatic component and the maximization of attractive contribution. Finally, Wheaton *et al.* [17] have suggested developing artificial nitro-substituted aromatic molecules as universal nucleobases.

In the present work, we use computational chemistry to study the stacking interactions between two types of nitrogen-substituted phenothiazine derivative. Information about the stacking contribution in a self-assembling process is difficult to isolate from experimental studies alone. Our calculations allow us to characterize the individual interactions between the nitro-substituted aromatic molecules, and thereby reveal the detailed contribution of different types of interactions giving a comprehensive picture of their stacking abilities.

## 2. Computational Details

For the computation of intermolecular interactions, local (L) electron correlation methods [18–20] at second order perturbation theory level have been proven to drastically reduce the computational effort and, at the same time, give values which are very close to the standard Møller-Plesset perturbation theory (MP2) results. By construction, this method is also virtually free of the basis set superposition error (BSSE) [19,20]. Quasi-linear scaling of the computational cost as a function of the system size [21] of the LMP2 method makes it possible to treat larger systems or to use larger basis sets. Using the density fitting (DF) approximation of the electron repulsion integrals [22–24] one can reduce again the computation time by about one order of magnitude, applying it both in Hartree-Fock (HF) and LMP2 cases (DF-HF and DF-LMP2). In this way, the computational cost is reduced to *O*(*N*)–*O*(*N*^2^) without losing much in accuracy compared with the case of the classical second-order Møller-Plesset perturbation theory (MP2), which scales formally with the order of *O*(*N*^5^). Furthermore, considering the local character of occupied and virtual orbitals in the local correlation treatment, one can easily obtain also the dispersion part (an intermolecular effect) of the correlation contribution [25].

In spite of the fact that the local correlation treatment combined with the density-fitting technique can, in general, provide calculations with much lower computational costs, the deficiency of the standard MP2 theory (overestimates the dispersion forces in π-stacked systems) remain also an attendant of the LMP2 method. Hill *et al.* [26] performed a detailed theoretical investigation for different dimer configurations of benzene and they found that the LMP2 results are quit far from the counterpoise corrected CCSD(T) values. At the same time, if they applied the so-called spin-component scaled (SCS) MP2 theory [27] (both for canonical and localized orbitals) the discrepancy was small compared with the CCSD(T) results. But, in their later work [28] they shown that in case of stacked nucleic acid systems the SCS-LMP2 method fails to describe correctly the intermolecular interaction energies, where its mean deviation from the best estimated values of the S22 set [29] is 1.62 kcal/mol. Accordingly, instead of the default scaling factor of 6/5 for antiparallel spins and 1/3 for parallel spins they completely neglect the contribution form antiparallel-spin electron pairs to the MP2 energy and scaled the parallel contribution by 1.76. In this way they obtained a mean deviation of −0.04 kcal/mol. The method is called spin-component scaled LMP2 for nucleobases (SCSN-LMP2). Choosing the spin-component scaling factors empirically, they accentuated that the methodology can no longer be considered a truly *ab initio* method, but it provides a substantial correction to the MP2 overestimation of the dispersion energy at no extra cost. At the same time, one should emphasized that, in case of benzene aromatic rings, no any direct calculation was found in the literature where the DF-SCSN-LMP2 and DF-LCCSD(T) methods were explicitly compared. On the other hand, the SCS values depend on the quality of the basis set. Accordingly, Distasio Jr and Head-Gordon developed a new SCS scheme, called SCS(MI) [30], which can overcome this deficiency and could gives intermolecular binding energies with quadruple-ζ accuracy.

The widely used semiempirically corrected DFT method by dispersion effects [31] adapted for several exchange-correlation (XC) functionals was also tested in our previous work [11]. We found that, practically there are no differences between the second-order local correlation potential curve and the values obtained with the semiempirically corrected DFT by dispersion effects considering the BLYP XC functionals (BLYP-D).

Due to the computer capacity limits, theoretical methods presented in the previous paragraph together with middle large basis sets are capable of handling molecular systems with approximately 150 atoms. At the same time, in the self-assembling processes are involved molecular systems, which all together contain more than 200–300 atoms. The density functional-based tight-binding method combined with the self-consistent charge technique (SCC-DFTB) [32] can be considered as an adequate solution for treating large biologically interested or nanoscaled molecular materials with nearly good accuracy as obtained in the case of high-level theoretical methods [33,34]. An empirical dispersion correction has also been developed, and it was found to be crucial for predicting reliable nucleic acid base stacking interactions [35], the relative stability of α and 3_10_ helices in proteins [36], and the stability of the double-stranded DNA tetramer with a ligand in the intercalative fashion [37].

Using the DF-LMP2 method implemented in the Molpro program package suite [38] we have performed geometry optimization for nitrogen substituted phenothiazine and their derivatives considering the cc-pVDZ basis set [39,40]. The intermolecular interaction energies and their dispersion components were obtained using the same method, but using a wide range of cc-pVNZ (where N = D,T) and aug-cc-pVNZ (where N = D,T) basis sets. Taking the program parameter descriptions as presented in Reference [26] we used the following input settings: (i) we have considered the Pipek-Mezey (PM) localization procedure [41]; (ii) in order to solve an occurring poor orbital localization in the PM technique when the larger diffuse basis set were used, we eliminate the contribution of the diffuse basis functions to the localization criteria by setting the corresponding rows and columns of the overlap matrix used in the PM localization to zero; (iii) the domains of the three aromatic π orbitals in each benzene-type ring were merged, leading to the three identical domains that include the p_π_ atomic orbitals of all 6 carbon atoms from the given six-membered ring. Molecular structures were visualized and analyzed using the open source Gabedit molecular graphics program [42].

## 3. Results and Discussion

### 3.1. Phenothiazine Dimers

**Figure 1.**
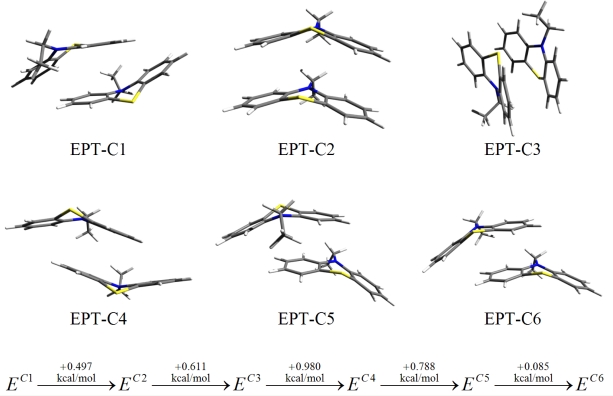
Conformational structures and their relative conformational energies for different ethyl-phenothiazine (EPT) dimers obtained at the DF-LMP2/cc-pVTZ level of theory. (See Reference [11]).

The intermolecular interaction of dimer structures of the phenothiazine derivatives were investigated in detail in our previous work [10]. Six different dimers of ethyl-phenothiazine (EPT) were found and their intermolecular binding was compared. The structures of these EPT dimers are presented in Figure 1. From the self-assembling point of view, we consider the EPT-C2 dimer as the appropriate conformation. We think, that only this conformation could maintain the long-range symmetry which is essential for a high-ordered self-assembling process.

**Figure 2.**
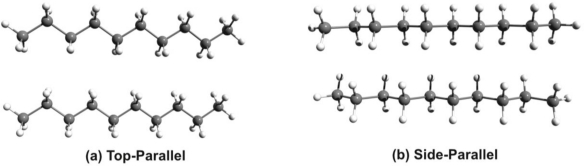
The side-parallel and top-parallel structures of the alkyl chain.

Comparing the conformational energy differences one can observe that the EPT-C2 structure shows a very good stability, being with only 0.497 kcal/mol above to the structure with the highest stability. Unfortunately, this close energetic proximity is not true for the alkyl chains. The difference between the most stable side-parallel structure and the top-parallel geometry (see Figure 2) which is required for the self assembling process could be relatively high, around 3–4 kcal/mol for nonane or decane alkyl chains.

Summarizing, the advantageous contribution of the EPT-C2 and the disadvantageous effect of the alkyl chain impose the use of alkyl chains shorter than hexane. In order to overcome the unavoidable destructive effect we changed two and four carbon atoms, respectively with nitrogen in the phenothiazine head-group. By this substitution the strength of the intermolecular interaction is enhanced by diminishing the destructive contribution of the alkyl chains.

### 3.2. Nitrogen-Substituted Phenothiazine Dimers

**Figure 3.**
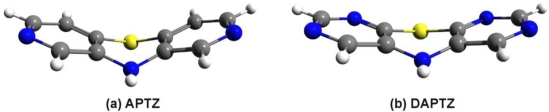
The optimized geometry structure of the azaphenothizaine (APTZ) and diazaphenothiazine (DAPTZ) molecules.

According to the statement drew in the previous subsection, we have figured out two different nitrogen substituted molecular structures, called azaphenothizaine (APTZ) and diazaphenothiazine (DAPTZ). Their molecular graphics is presented in Figure 3. For these two molecular structures we have performed geometry optimization using the DF-LMP2 method with cc-pVDZ basis set.

One of the conclusion drew up in our previous work [11] was that considering the cc-pVDZ basis set one can obtain adequate intermolecular distances and geometry structure compared with larger basis sets, as cc-pVDZ, cc-pVQZ or their augmented form. It is not the case for the intermolecular interaction energies, where large discrepancies were found for different basis sets. For this, one needs to use at least triple-ζ quality basis sets. Accordingly, to establish the correct intermolecular interaction energies in the APTZ and DAPTZ dimers we have considered the wide range of cc-pVNZ (where N = D, T) and aug-cc-pVNZ (where N = D, T) basis sets.

**Figure 4.**
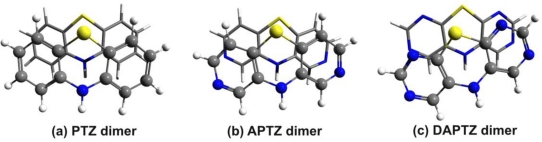
The optimized geometry structure of the azaphenothizaine (APTZ) and diazaphenothiazine (DAPTZ) dimers.

The energy results are collected in Table 1, while the molecular graphics of the APTZ and DAPTZ dimers is presented in Figure 4. Compared the relative position of the monomers, which has a totally symmetric form for the PTZ dimer, in case of nitrogen-substituted systems the monomers are rotated with 8.4° for APTZ and with 16.7° for the DAPTZ dimer.

**Figure 5.**
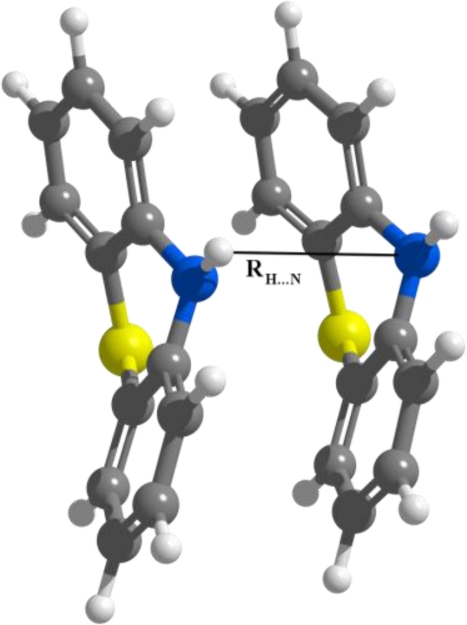
The intermolecular *R*_H…N_ coordinate in the PTZ dimer.

We consider that in the first case of APTZ the relative rotation of the monomers is not so significant in order to destroy the predisposition for the self-assembling, but in the second case this rotation could be large enough to break the self-assembling. In a later section we have investigated the probability of this assumption by adding alkyl chains to the nitrogen-substituted head-groups. Comparing the d(N···H) distances (measured between the N atom of the first monomer and the H atom belonging to the N–H bond of the second monomer—see Figure 5) for all three monomers (PTZ, APTZ and DAPTZ) we have found that the shortest distance is obtained for the APTZ dimer (d(N···H)^APTZ^ = 3.557 Å). The distance d(N···H)^DAPTZ^ increase to 3.598 and reach 3.665 Å for the PTZ dimer.

The nature of the intermolecular energies was analyzed at HF and correlation level of theories. The correlation energy was decomposed in two parts, defined in the framework of the electron correlation theory taking into account the local character of occupied and virtual orbitals [25]. Analyzing the energy values presented Table 1, one can observe that, similarly to the PTZ case, the intermolecular interaction is exclusively stabilized by the electron correlation effects. For example, in case of the APTZ dimer the interaction energy obtained at HF level with aug-cc-pVTZ basis set is Δ*E*^HF^ = +10.024 kcal/mol. This strong electrostatic repulsion is canceled by a stronger attractive force, mostly given by the dispersion effects (*E*^Disp.^ = −20.378 kcal/mol). Similar situation can be found for DAPTZ dimer. Here, the HF/aug-cc-pVTZ energy is Δ*E*^HF^ = +9.887 kcal/mol, while the electron correlation part is Δ*E*^Corr^ = +24.834 kcal/mol.

**Figure 6.**
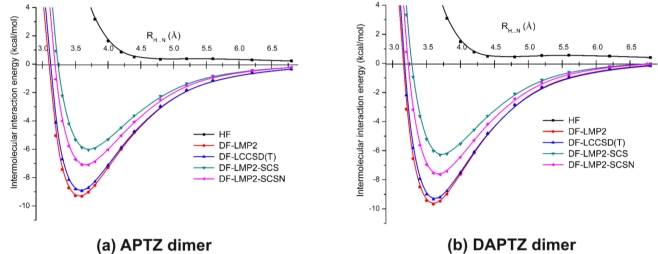
The potential energy curves for azaphenothizaine (APTZ) and diazaphenothiazine (DAPTZ) dimers obtained at different theoretical methods and using the cc-pVDZ basis set.

Figure 6 shows a comparison of the potential energy curves for APTZ (a) and DAPTZ (b), obtained at HF and electron correlation levels of theory, using the cc-pVDZ basis set. The energy minimum and the corresponding equilibrium distance for each curve presented in Figure 6 are given in Table 2.

In the case of electron correlation calculations the conventional MP2, CCSD(T) and two spin component scaling MP2 methods were considered together with the density-fitting and orbital localization approximations. Considering any of the dimer case, it can be seen that HF curves do not give bounded states, while the potential curves drawn with the help of electron correlation methods show energy minima.

Assuming the fundamental idea of Grimme [27] (where the spin component scaling was introduced), methods based on this idea (DF-LMP2-SCS and DF-LMP2-SCSN) give weaker non-covalent bonds, than those obtained for methods based on the conventional electron correlation theories (DF-LMP2 and DF-LCCSD(T)). The energy differences are in the range of 1.8–3.3 kcal/mol.

**Figure 7.**
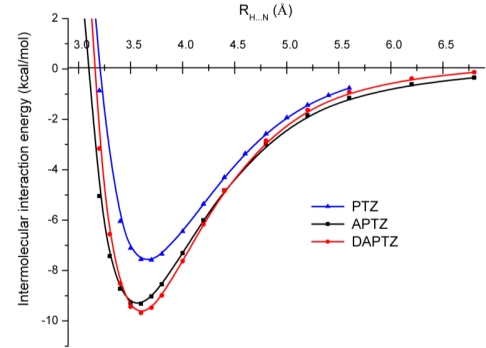
The potential energy curves for phenothiazine (PTZ), azaphenothizaine (APTZ) and diazaphenothiazine (DAPTZ) dimers obtained at DF-SCSN-LMP2 theoretical method and using the cc-pVDZ basis set.

A similar conclusion was obtained also in the case of phenothiazine dimers [11]. In order to be able to discern the effect of the substituted nitrogen atoms in the phenothiazine we have put together (See Figure 7.) the three potential energy curves obtained at DF-SCSN-LMP2 level of theory, applying the cc-pVDZ basis set. As we can see, the black and red curves specific for APTZ and DAPTZ pairs show dimers that are stronger with ≈2.0 kcal/mol than the PTZ dimer (*blue* curve).

### 3.3. SCC-DFTB Results

In order to obtain a comprehensive picture of the self-assembling process, one needs to treat the *functional group*, *spacer*, and *linker* all together and should also consider a suitable large number of monomers. These requirements could easily exceed the efficiency limit of any conventional *ab initio* method (HF, MP2, DFT, *etc*.). Therefore, we need to consider theoretical methods (including both intra- and intermolecular effects) which can easily treat molecular systems with a large number of atoms, but at the same time able to give results comparable with *ab initio* methods. In this respect, the density functional-based tight-binding method combined with the self-consistent charge technique (SCC-DFTB) [32] can provides the suitable framework to study larger molecular systems, due to the empirical dispersion correction which is included in the theory.

To make sure about the efficiency of the SCC-DFTB method in our earlier work [11] we have performed a comparative study considering the DF-SCSN-LMP2/aug-cc-pVTZ, PM6-DH2 semiempirical [43] and MM3 force field [44] methods. Our conclusion was that the SCC-DFTB method gives values close to the PM6-DH2 quality, having a better long-range behavior, but a poorer short-range match with the potential curve of the reference *ab initio* method.

As first step we built dimer structures for both APTZ and DAPTZ molecular species considering for the spacer butane and nonane alkyl chains and a thiol fragment as linker. The starting geometries are based on the equilibrium positions of the two molecular species and the spacer and linker groups were subsequently added.

**Figure 8.**
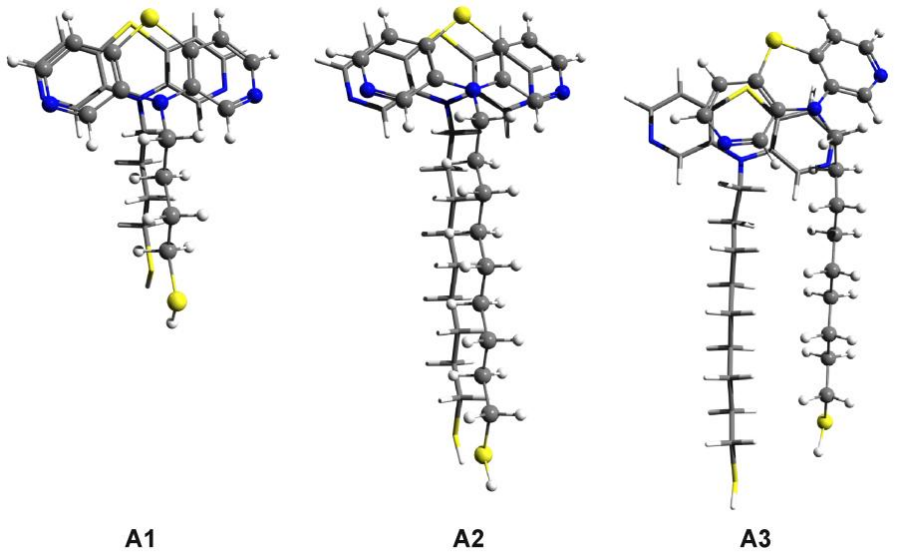
The optimized dimer geometries for *thiol-butyl-APTZ* (**A1**) and for two configurations of *thiol-nonyl-APTZ* (**A2** and **A3**) obtained at SCC-DFTB level of theory.

The optimized dimer geometries for *thiol-butyl-APTZ* (A1) and for two configurations of *thiol-nonyl-APTZ* (A2 and A3) are presented in Figure 8. In the first two cases (A1 and A2) a highly-ordered dimer structure characteristic for the high efficiency self-association were obtained. The functional groups show the EPT-C2 type configuration, while the alkyl chains pack in the top-parallel configuration (See Reference [11]). The intermolecular energies are: −13.67 kcal/mol for the *thiol-butyl-APTZ* and −17.02 kcal/mol in case of *thiol-nonyl-APTZ*. We have calculated also the deformation energy induced by the dimer association which is: −0.2 kcal/mol for the *thiol-butyl-APTZ* and −0.27 kcal/mol in case of *thiol-nonyl-APTZ*. These deformation energies are quite small which means that the monomer distortions can be neglected in case of the high-ordered molecular association. In order to assess the stability of the highly-ordered structure we have built some geometries specific to a hypothetic “defected dimer”. Here, we call “defected dimer” those structures which are not packed in the highly-ordered form (EPT-C2 for the head group and top-parallel for the alkyl chain). The optimized geometry of such defected dimer of *thiol***-***nonyl***-***APTZ* can be seen in Figure 8/A3. The intermolecular interaction energy is −20.08 kcal/mol, slightly lower that for the highly-ordered structure. In addition, we have computed also the energy difference between the ordered (A2) and the “defected” (A3) configurations as well as the energy of the monomer deformation (A3).

**Figure 9.**
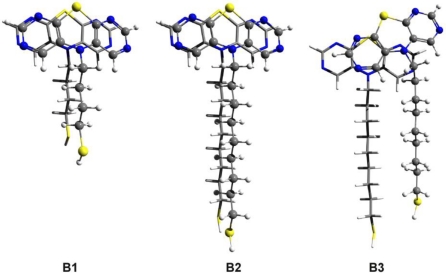
The optimized dimer geometries for *thiol-butyl-DAPTZ* (**B1**) and for two configurations of *thiol-nonyl-DAPTZ* (**B2** and **B3**) obtained at SCC-DFTB level of theory.

The values are: 1.49 kcal/mol and 1.84 kcal/mol, respectively. As we can see, there is a significant amount of deformation energy which can weaken the intermolecular interaction and accordingly decrease the conformational energy difference between the ordered and the “defected” structures. Similar molecular geometries were built in the case of DAPTZ-type head-group. The molecular structures with optimized geometry are shown in Figure 9. Similar ordered and defected configurations were obtained for *thiol-butyl-DAPTZ* (B1) and *thiol-nonyl-DAPTZ* (B2 and B3). The intermolecular energies of the ordered structures are: −12.78 kcal/mol for the *thiol-butyl-APTZ* and −16.11 kcal/mol *thiol-nonyl-APTZ*, respectively. The interaction energy for the *thiol-butyl-APTZ* “defected” geometry is −18.87 kcal/mol. On the same grounds, the configuration energy difference between the ordered (B2) and the “defected” (B3) configurations is 1.29 kcal/mol, while the monomers deformation energy (B3) is 1.79 kcal/mol.

Comparing energies corresponding to similar structures, one can conclude that the substitution of the second set of nitrogen atoms does not increase the efficiency of the dimer association. In both cases of *thiol-butyl-DAPTZ* and *thiol-nonyl-DAPTZ* the interaction energies are smaller than those corresponding to the similar structures with APTZ-type head-groups. On the other hand, a comparative analysis of the structures with APTZ-type and PTZ-type head-groups, suggests two main statements. The presence of the first set of nitrogen atoms are able to favor highly-ordered self-assembled supramolecular structures due to increase of the π–π stacking interaction between the functional (head-) groups. However, their presence is not able to increase the selectivity of the highly-ordered process, because similar an increase of the head-group interaction appears in all configurations presented in Figure 1.

## 4. Conclusions

The intermolecular π–π stacking interaction between some PTZ derivatives having attached alkane chains with different lengths have been investigated and described quantitatively in a previous paper [11]. The present contribution describes similar π–π stacking interaction between two types of nitrogen substituted PTZ derivatives having a stronger tendency to self-organize in supramolecular structures. Similar hypothetical parallel and antiparallel stacking conformations were investigated in both cases and stacking structures joining together in a characteristic “V” shape have been found to be the most stable supramolecular architectures. It was found that the conformational stability of these molecular systems is ensured by the dispersion-type electron correlation effects. Considering the LMP2 geometry, in all cases, the HF contribution in the intermolecular interaction energy gives positive values, bringing an unfavorable contribution to the dimmer stability. This effect is compensated by the larger contribution of dispersion forces, which finally keeps together the stacking structures. As revealed the obtained results, the net effect brought by the nitrogen atoms is to increase the binding tendency and to enhance the dimmers stability. If the potential energy curves of nitrogen substituted and non-substituted phenothiazine derivatives are compare one can see that the difference between the energetic minima is about 2 kcal/mol. Accordingly, our prediction is that the process of self-assembling (in monolayers for example) having as the basic unit some nitrogen substituted PTZ derivatives might occur with a higher probability. In terms of associations efficiency one can estimate that, at room temperature, the association constant for the nitrogen substituted molecular species increases with about one order of magnitude as compared to non-substituted case. Unfortunately, similar energetic arguments must be considered for the structures from the sequence EPT-C1···C6, the direct consequence being that there remain a lot of “defected” structures with energies a little bit higher but with values that are relatively close to the most stable structure. Furthermore, several changes seem to be appropriate to increase even more the self-assembling ability of the nitrogen substituted PTZ derivative.

**Figure 10.**
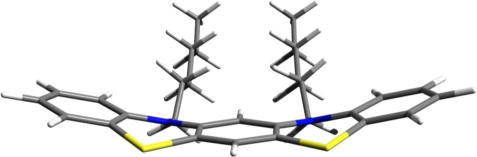
The hypothetically designed structure obtained by partial superposition of two PTZ units with two alkyl chains attached to nitrogen atoms.

For instance, two rings from two neighboring PTZ molecules could be coupled together in a molecular structure with five rings and two alkyl chains with variable length (See Figure 10). Our guess is that additionally to the enhancement of the π–π stacking interaction the hypothetically designed structure described above will be able to self-assemble in more ordered supramolecular structures.

## Figures and Tables

**Table 1.** Intermolecular interaction energies and their characteristic components (dispersion and ionic) defined in the framework of the LMP2 theory obtained at HF and LMP2 levels of theory and different basis sets.

	**Δ*E*^HF^ (kcal/mol)**	**Δ*E*^DF-LMP2^ (kcal/mol)**	***E*^Corr.^ (kcal/mol)**	***E*^Disp.^ (kcal/mol)**	***E*^Ion.^ (kcal/mol)**
**APTZ**					
cc-pVDZ	+7.056	−9.343	−16.399	−13.854	−3.462
cc-pVTZ	+9.307	−12.322	−21.629	−17.741	−5.312
aug-cc-pVDZ	+7.136	−15.245	−22.381	−19.777	−6.723
aug-cc-pVTZ	+10.024	−15.138	−25.162	−20.378	−6.117

**DAPTZ**					
cc-pVDZ	+6.445	−9.696	−16.141	−14.044	−3.625
cc-pVTZ	+8.992	−12.338	−21.330	−17.806	−5.485
aug-cc-pVDZ	+7.616	−14.714	−22.330	−19.807	−6.926
aug-cc-pVTZ	+9.887	−14.947	−24.834	−20.397	−6.232

**Table 2.** The *R*_H…N_ equilibrium distances and the corresponding intermolecular interaction energies for azaphenothizaine (APTZ) and diazaphenothiazine (DAPTZ) dimers obtained at different theoretical methods and using the cc-pVDZ basis set.

	**LMP2**	**LCCSD(T)**	**LMP2-SCS**	**LMP2-SCSN**
**APTZ**				
*R*_e_ (Å)	3.564	3.585	3.700	3.656
*E*_e_ (kcal/mol)	−9.287	−8.888	−5.999	−7.097

**DAPTZ**				
*R*_e_ (Å)	3.605	3.622	3.728	3.680
*E*_e_ (kcal/mol)	−9.606	−9.277	−6.257	−7.603
